# Preoperative endoscopic marking of the gastrointestinal tract using fluorescence imaging: submucosal indocyanine green tattooing versus a novel fluorescent over-the-scope clip in a survival experimental study

**DOI:** 10.1007/s00464-020-07999-2

**Published:** 2020-09-28

**Authors:** Manuel Barberio, Margherita Pizzicannella, Andrea Spota, Anila Hoskere Ashoka, Vincent Agnus, Mahdi Al Taher, Boris Jansen-Winkeln, Ines Gockel, Jacques Marescaux, Lee Swanström, Seong-Ho Kong, Eric Felli, Andrey Klymchenko, Michele Diana

**Affiliations:** 1 IHU-Strasbourg, Institute of Image-Guided Surgery, 1, place de l’Hôpital, 67091 Strasbourg, France; 2 IRCAD, Research Institute Against Digestive Cancer, Strasbourg, France; 3 Laboratoire de Bio-Imagerie et Pathologies, UMR 7021 CNRS, Université de Strasbourg, Strasbourg, France; 4 Department of Visceral, Transplant, Thoracic and Vascular Surgery, University Hospital of Leipzig, Leipzig, Germany; 5 Department of Surgery, Seoul National University, Seoul, South Korea

**Keywords:** Fluorescence-guided surgery, Preoperative endoscopic marking, Preoperative endoscopic tattooing, Fluorescent over the scope clip, Indocyanine green tattooing

## Abstract

**Background:**

Intraoperative localization of endoluminal lesions is can be difficult during laparoscopy. Preoperative endoscopic marking is therefore necessary. Current methods include submucosal tattooing using visible dyes, which in case of transmural injection can impair surgical dissection. Tattooing using indocyanine green (ICG) coupled to intraoperative near-infrared (NIR) laparoscopy has been described. ICG is only visible under NIR-light, therefore, it doesn’t impair the surgical workflow under white light even if there is spillage. However, ICG tattoos have rapid diffusion and short longevity. We propose fluorescent over-the-scope clips (FOSC), using a novel biocompatible fluorescent paint, as durable lesion marking.

**Methods:**

In six pigs, gastric and colonic endoscopic tattoos using 0.05 mg/mL of ICG and markings using the fluorescent OSC were performed (T0). Simultaneously, NIR laparoscopy was executed. Follow-up laparoscopies were conducted at postoperative day (POD) 4–6 (T1) and POD 11–12 (T2). During laparoscopy, fluorescence intensity was assessed. In one human cadaver, FOSC was used to mark a site on the stomach and on the sigmoid colon, respectively. Intraoperative detection during NIR laparoscopy was assessed.

**Results:**

Gastric and colonic ICG tattooing and OSC markings were easily visible using NIR laparoscopy on T0. All FOSC were visible at T1 and T2 in both stomach and colon, whereas the ICG tattooing at T1 was only visible in the stomach of 2 animals and in the colon of 3 animals. At T2, tattoos were not visible in any animal. FOSC were still visible in both stomach and colon of the human cadaver at 10 days.

**Conclusion:**

Endoscopic marking using FOSC can be an efficient and durable alternative to standard methods.

**Electronic supplementary material:**

The online version of this article (10.1007/s00464-020-07999-2) contains supplementary material, which is available to authorized users.

Accurate localization of endoluminal lesions of the gastrointestinal (GI) tract is a fundamental part of resectional GI surgery. Since neither laparoscopic nor robotic surgery provides tactile feedback, it is impossible to identify lesions by palpation and the surgeon must rely on visual assessment or, to some extent, on preoperative imaging alone. GI lesions can also be identified using intraoperative endoscopy, but this additional procedure adds to the overall surgery time and can cause bowel distension, which may impair the operative field for the surgeon [1]. This has led to preoperative endoscopic marking of intraluminal lesions, invisible from the serosal side, being an essential part of minimally invasive GI resection procedures. The endoscopic injection of India ink in the submucosal space (tattooing) was first described in 1975 [2]. Although this dye produces a permanent and easily visible staining, complications such as abscess formation, focal peritonitis [3, 4], inflammatory tumors [5], idiopathic inflammatory bowel disease [6], or adhesion-related ileus [7] have been described. For this reason, a safer and equally efficient carbon-based sterile dye, the SPOT™ (GI Supply, USA) was commercialized [8]. A common drawback of both these dyes is that, when inadvertent transmural injection occurs (reported incidence 2.4–13%), substantial peritoneal staining may occur [9]. This, in turn, can conceal surgical dissection planes making surgery more challenging, time-consuming, and potentially dangerous [10]. Although the reported rate of accidental extraperitoneal contamination during endoscopic tattooing is low, this still means that a substantial number of patients will have this complication, considering that 1.8 million new cases of colorectal cancer were estimated in 2018 according to the GLOBOCAN database of the World Health Organization, [11].

An alternative to tattooing is the endoscopic placement of endoclips adjacent to the lesion, which can then be localized using intraoperative fluoroscopy. However, the use of a C-arm in the operating room (OR) has a negative impact on the surgical workflow and exposes both patient and staff to ionizing radiations. Additionally, endoclips have a substantial rate of spontaneous detachment from the he mucosa (after 10 days on average), and this can jeopardize the identification of the lesion during surgery [12].

India ink, SPOT™, and endoclips, which are the most commonly used methods in clinical practice to mark GI lesions preoperatively, therefore suffer from considerable limitations. This has motivated researchers to seek more efficient alternatives. Recently, preoperative endoscopic tattooing of gastric [13, 14] and colorectal [15–18] lesions using indocyanine green (ICG), an exogenous fluorophore visible in real time using near-infrared (NIR) cameras, has been described. ICG is a cyanine dye, which is a brilliant visible green at high concentrations, but remains invisible at lower concentrations. When excited with a NIR light source, it emits a fluorescent signal, which can be visualized using cameras able to detect wavelengths within the NIR spectrum. NIR fluorescence imaging is a sensitive technique with a good spatial resolution and an in vivo tissue penetration of up to 10 mm [19]. Such cameras can typically operate in both white light and NIR mode and are increasingly available in laparoscopic ORs. The invisibility of low-concentration ICG under white light overcomes the main limitation common to all visible tattooing agents, the obscuring of surgical dissection planes in cases of peritoneal spillage. The main problem with submucosal ICG tattooing is that it diffuses relatively rapidly and tends to disappear within 7 to 10 days [17].

Recently, our group has developed a biocompatible fluorescent paint with similar wavelength sensitivities but an increased intensity as compared to ICG [20, 21]. This fluorescent agent can coat any metallic or plastic device and when excited with NIR light, it emits a powerful fluorescent signal.

We hypothesized that commercially available over-the-scope clips, once coated with the fluorescent paint, could serve as effective and durable marker for GI lesions that could be readily seen with today’s NIR equipped laparoscopic cameras. The aim of the current study was to compare the intraoperative detection rate of gastrointestinal ICG tattooing and marking with a fluorescent-coated over-the-scope clip (FOSC).

## Methods

### Fluorescently coated clips

The fluorescent coating material (near-infrared coating of equipment: NICE) was synthesized by combining a biocompatible polymer poly(methyl methacrylate) (PMMA) with a specifically engineered fluorescent dye, which displays optical properties rather similar to ICG, but with a higher brightness and stability [20–22]. The fluorescent paint can coat medical instruments using direct immersion or a paintbrush. After drying for 30 min, the coated instrument is ready to use. Twelve 11 mm nitinol OSC with hexagonal shape and six inner prongs (Padlock Clip™, US Endoscopy, United States) were coated three times by means of direct immersion one day before the procedure.

### Porcine model experiment

#### Animals

This study is part of the ELIOS protocol (Endoscopic Luminescent Imaging for Oncology Surgery), approved by the local Ethical Committee on Animal Experimentation (ICOMETH No. 38.2016.01.085), and by the French Ministry of Superior Education and Research (MESR), (APAFIS#8721–2017013010316298-v2). No informed consent nor IRB approval were needed since this is an experimental study.

Six adult pigs (Sus scrofa domesticus, ssp. Large White, mean weight: 32.3 ± 7.5 kg; 3 females) were involved in the survival study. One additional animal, recovered after being used for training purposes in our surgical training facility, was included and served to assess the preliminary feasibility of the procedure. All animals used in the experimental platform were handled according to French laws for animal use and care, to the directives of the European Community Council (2010/63/EU) and ARRIVE guidelines [23]. A bowel preparation was started 48 h before surgery by administering 2 L of Colopeg (Bayer, Germany). All animals had free access to water. Intramuscular ketamine (20 mg/kg) and azaperone (2 mg/kg) (Stresnil, Janssen-Cilag, Belgium) were used for premedication. Induction was achieved with intravenous propofol (3 mg/kg) combined with rocuronium (0.8 mg/kg). Anesthesia was maintained with 2% isoflurane. From postoperative day (POD) 1, the animals received a standard semi-solid diet. The total length of the survival period was 12 days. A control laparoscopy (T1) was performed either on POD 6 (*n* = 3) or POD 4 (*n* = 3). On POD 12, the animals underwent a further control laparoscopy (T2) and were sacrificed with an intravenous injection of pentobarbital sodium (40 mg/kg) (Exagon®, AXIENCE, France), under a 5% isoflurane anesthesia.

#### Endoscopic marking/tattooing, surgical procedure, and follow-up

On day 0 (T0), each animal had two endoluminal fluorescent clips placed and two 1 mL volume ICG tattoos. Interventions were performed in the stomach and in the rectosigmoid colon respectively. Both organs were divided into three regions: in the stomach: 1 = antrum, 2 = body and 3 = fundus, in the colon 1 = distal sigmoid, 2 = mid-sigmoid, 3 = proximal sigmoid, and each intervention was randomly performed in only one region of each organ (Table 1). A laparoscopy was next performed using three ports (two 10-12 mm and one 5 mm) and a NIR laparoscope (D-Light-P, KARL STORZ, Germany). This camera can be switched from white light to NIR mode, using a footswitch, which activates the NIR light source and a bypass filter concurrently. A simultaneous gastroscopy was performed with a 9.3 mm gastroscope (Silver Scope®, KARL STORZ, Germany). Using a laparoscopic Babcock grasper, the first part of the duodenum was clamped, to avoid bowel loop distension. Within the previously assigned region, a spot on a portion of the anterior wall was chosen, and the endoscopic tattooing was performed using an endoscopic 0.5 mm injection needle (Interject™, Boston Scientific, United States). The mucosa was lifted by injecting 0.5 mL of distilled water in the submucosal space, followed by 1 mL of ICG (0.05 mg/mL) and a final push of 0.5 mL of distilled water, empty the needle from the remnant ICG. In addition, the tattooed area was marked laparoscopically with a 2/0 Vicryl suture (Ethicon, United States).

**Table 1:** Main results

Pig (N, Sex)	T1 POD	Stomach							Rectum							Complications
		ICG				Clip			ICG				Clip			
		Site	Spillage	Visibility		Site	Visibility		Site	Spillage	Visibility		Site	Visibility		
				T1	T2		T1	T2			T1	T2		T1	T2	
1 M	6	1	No	No	No	3	Yes	Yes	1	No	No	No	3	Yes	Yes	No
2 M	6	2	Yes	No	No	1	Yes	Yes	2	Yes	No	No	1	Yes	Yes	Small wound abscess
3 F	6	3	No	No	No	2	Yes	Yes	3	No	No	No	2	Yes	Yes	Sepsis due to small bowel perforation
4 F	4	3	No	Yes	No	1	Yes	Yes	3	Yes	Yes	No	1	Yes	Yes	No
5 F	4	1	No	Yes	No	3	Yes	Yes	1	Yes	Yes	No	3	Yes	Yes	No
7 M	4	1	No	No	No	2	Yes	Yes	3	No	Yes	No	1	Yes	Yes	No

Table describing the main results of the study, comparing the ICG tattooing with the over-the-scope clip as preoperative endoscopic marking in the stomach and in the rectum

*N* number, *M* Male, *F* female, POD post-operative day, *T1* control laparoscopy 1; *T2* control laparoscopy 2

The fluorescently coated clip was then loaded inside a Lock-It™ delivery system (US Endoscopy, United States), using a custom-made loading device and mounted at the distal tip of the gastroscope. Once in the stomach, an easily visible spot on the anterior wall within the randomly assigned region was identified and the FOSC was released by pushing the release button while suctioning the mucosa into the cap.

After clamping the colon as high as possible, to minimize bowel distension, ICG tattooing and the fluorescent OSC were applied endoscopically within the assigned regions and under laparoscopic guidance, in a similar fashion as above.

For intensity measurments, an ICG reference card (Diagnostic Green GmbH, Germany) was placed intra-abdominally next to each gastric and rectal tattoo and clip respectively, and a short video clip in NIR mode was recorded. The reference card was retrieved, and the animals returned to the animal keeping facility. A fluorescence intensity analysis (explained in the following paragraph) was performed postoperatively.

In three animals, the first follow-up, T1 was performed on POD 4 and in three animals on POD 6. The second control laparoscopy, T2 was performed on POD 11 (*n* = 1) and POD 12 (*n* = 5). During T1 and T2, the fluorescence signal analysis was repeated.

After T2, animals were humanely sacrificed, and the marked areas were harvested for measurements and gross inspection.

#### Software-based fluorescence analysis

The videos of ICG tattoos, clip markings and adjacent reference cards at T0, T1, T2 were analyzed postoperatively using a proprietary intensity measurement software. Since the fluorescence signal is inversely related to the distance between light source and target object [24], we corrected for this bias by calculating the relative fluorescence intensity, i.e., the ratio between the target and the adjacent reference card’s fluorescence intensity. The fluorescence intensity was defined in arbitrary units (a.u.). Additionally, to the software-based analysis, a subjective image analysis was carried out. Three authors (MB, MP, AS) performed a classification of all images in two categories: visible (Y) and not visible (N). The mean of the fluorescence intensity of each image and the binary classification (visible fluorescence/non-visible fluorescence) were analyzed using logistic regression.

### Human anatomical specimen experiment

One male human torso was used for this experiment. A FOCS was applied endoscopically in correspondence of the rectosigmoid junction and one in correspondence of the anterior gastric wall, a simultaneous laparoscopy was performed to assess real-time NIR clip visualization.

#### Sample size calculation and statistical analysis

The sample size was calculated based on data available in the literature. The primary outcome was the visibility of the submucosal ICG tattooing vs. the FOSC. Considering that submucosal ICG tattooing is no longer visible after 12 days (0%) [17], we hypothesized that at least 80% of the FOSC would still be visible. Using a superiority design for binary outcomes, five tests per group (total of 10 tests) are required to have a 95% chance of detecting, as significant at the 1% level, an increase from 0% in the control group (ICG) to 80% in the experimental group (FOSC). Values are reported as mean ± SD. Statistics were performed using the Prism 8 software (Graph Pad Software, United States). One-way ANOVA with Tukey’s multiple comparison test was used to compare the fluorescence intensity at the different time points. Logistic regression was performed to calculate the human visibility threshold of the fluorescence intensity.

A *p* value < 0.01 was considered statistically significant.

## Results

### Porcine model experiment

The main results are schematically reported in Table 1.

Mean weight was 34 ± 8.3 kg at T1 and 34.6 ± 9 at T2, the mean thickness of the stomach and of the rectum was 5 ± 2.83 mm and 2.46 ± 1.3 mm, respectively. In one animal (pig #3), T2 was performed one day earlier (POD 11 instead of POD 12) since the animal showed signs of sepsis, subsequently found to originate from a small bowel perforation. The perforation was remote from any tattooing/clipping marking area, and probably occurred during control laparoscopy at T1 from a laparoscopic grasper injury. One animal developed a small wound abscess at a 10 mm port site, which was successfully drained at T1. In the rest of the animals, there were no complications.

**Fig. 1:**
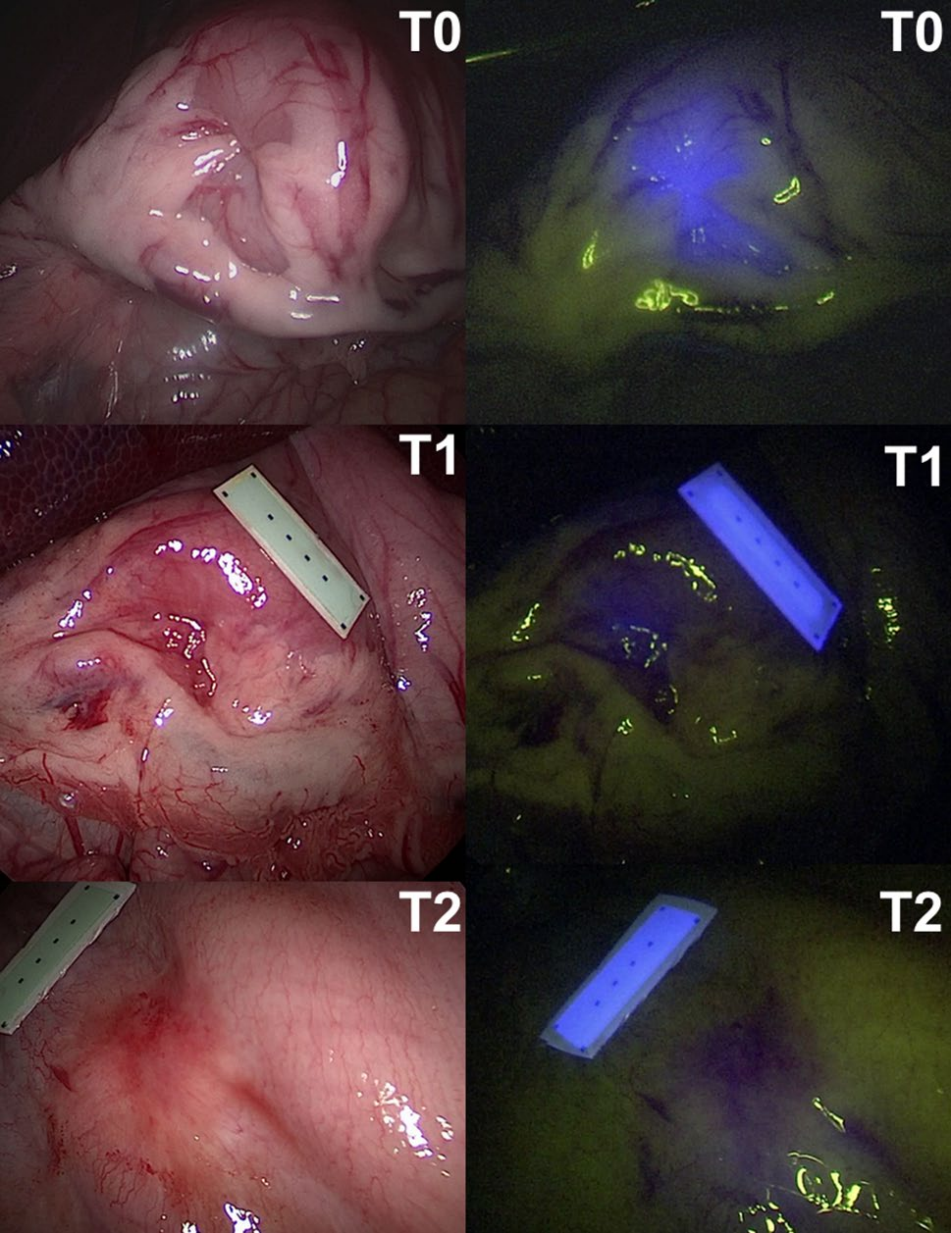
Gastric fluorescent clip marking in the porcine model. White light (left) and NIR imaging (right) mode visualization of the FOSC at three different time points (T0 = immediately after placement; T1 = postoperative day 6; T2 = postoperative day 12)

**Fig. 2:**
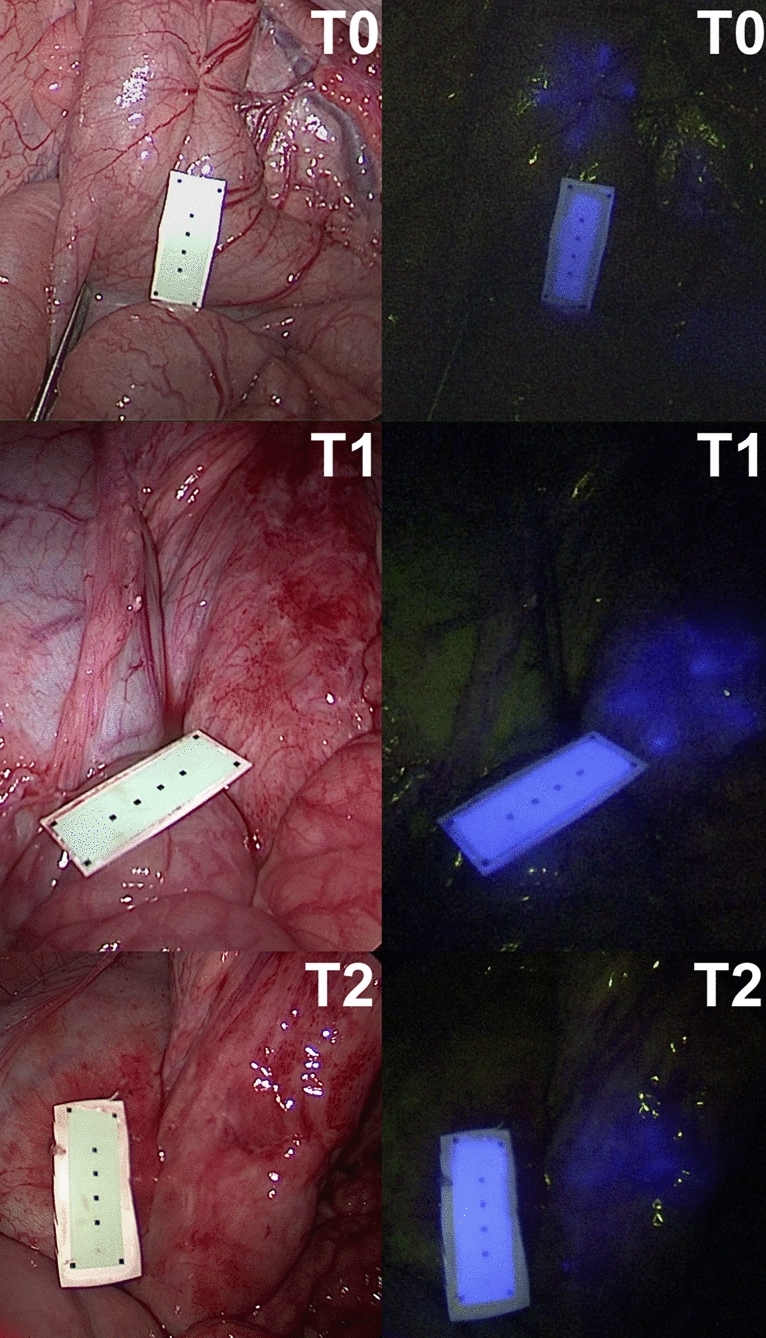
Colonic fluorescent clip marking in the porcine model. White light (left) and NIR imaging (right) mode visualization of the FOSC at three different time points (T0 = immediately after placement; T1 = postoperative day 6; T2 = postoperative day 12)

**Fig. 3:**
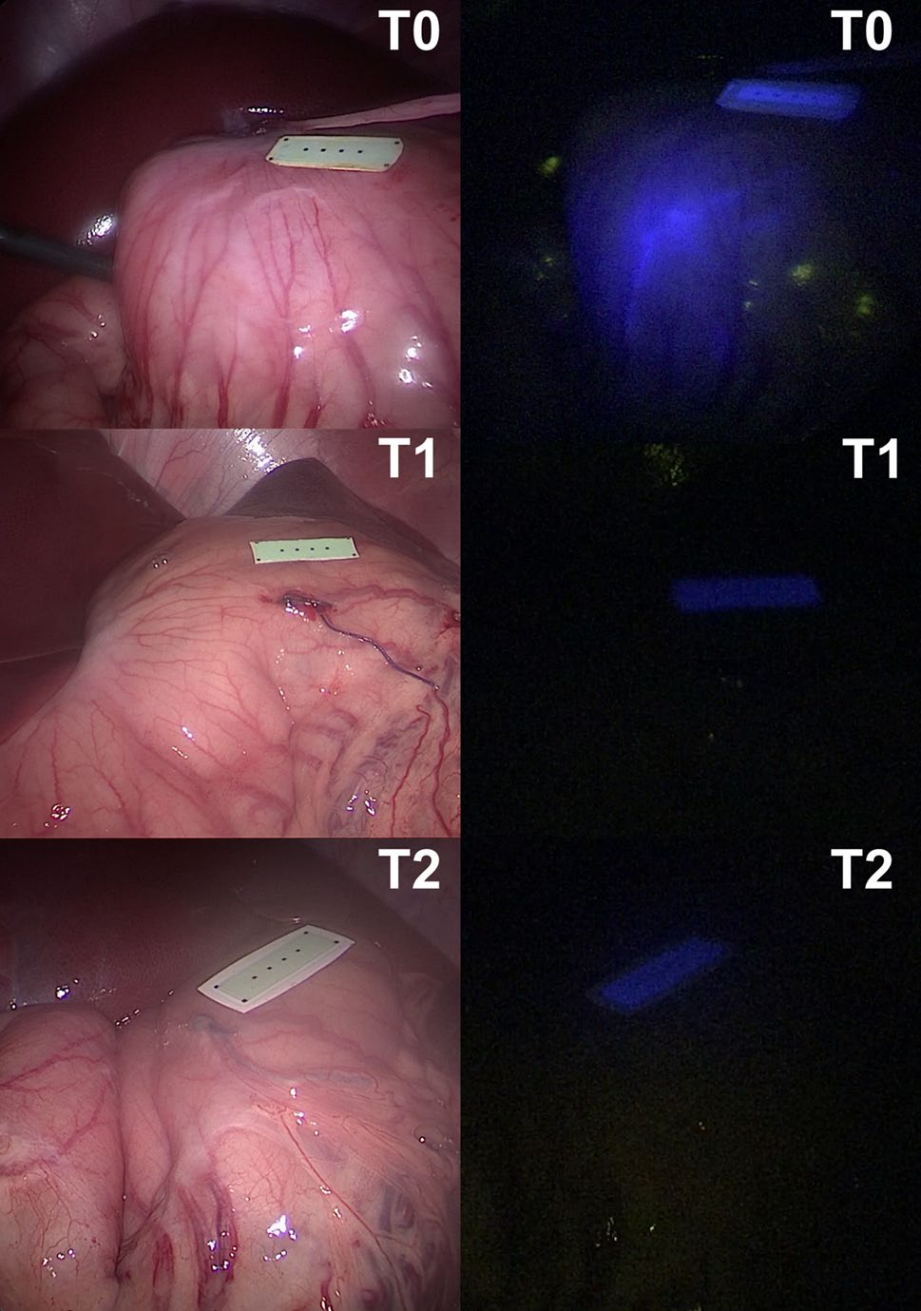
Gastric ICG tattooing in the porcine model. White light (left) and NIR imaging (right) mode visualization of the ICG tattooing at three different time points (T0 = immediately after placement; T1 = postoperative day 4; T2 = postoperative day 12)

**Fig. 4:**
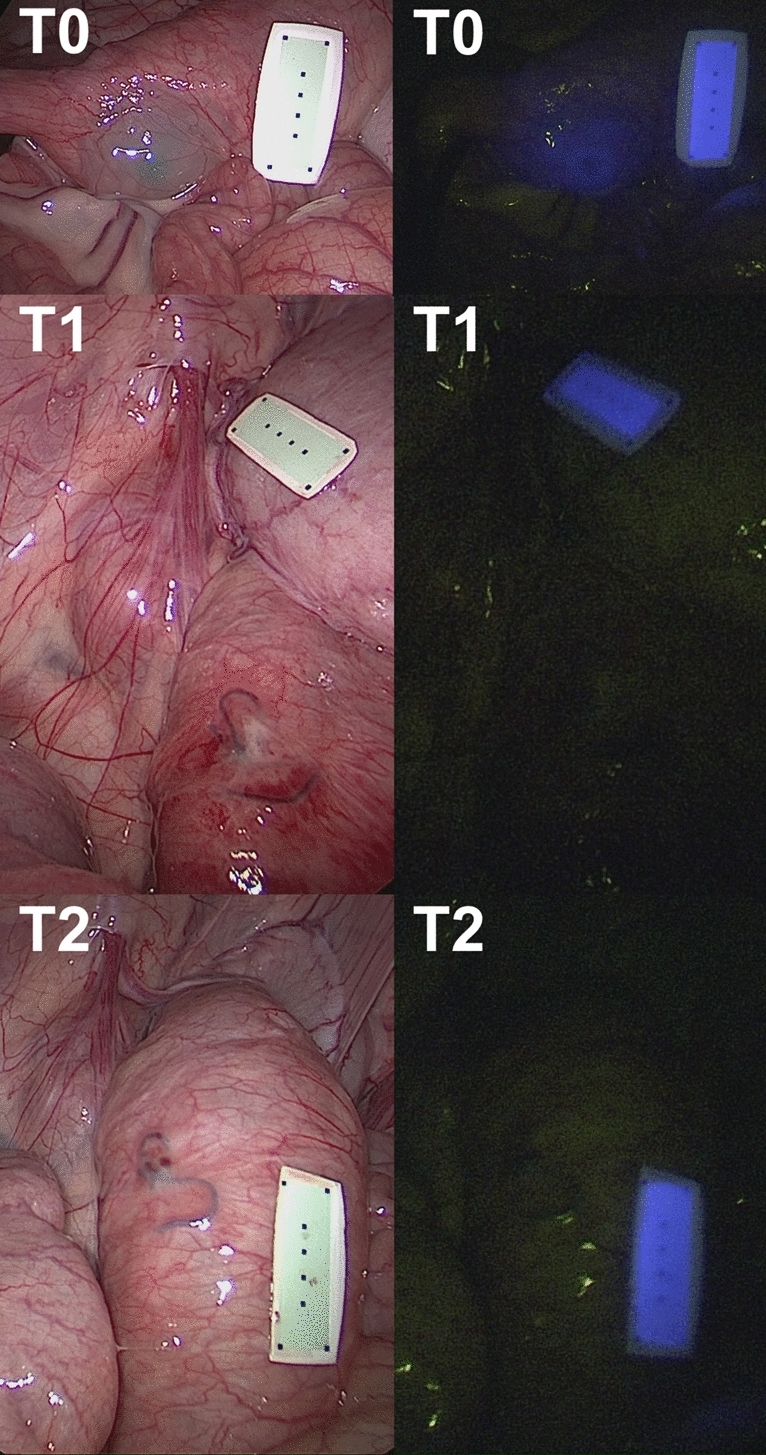
Colonic ICG tattooing in the porcine model. White light (left) and NIR imaging (right) mode visualization of the ICG tattooing at three different time points (T0 = immediately after placement; T1 = postoperative day 4; T2 = postoperative day 12)

In all animals, gastric and colonic ICG tattooing and clip markers were easily visible with fluorescence imaging on POD 0 (Figs. 1, 2, 3, 4 and videoclip). All clip markers were visible at T1 and T2 in both stomach and colon, whereas ICG tattooing at T1 was only visible in the stomach of two animals and in the colon of three animals. At T2, ICG tattoos were not visible in any animal.

### Fluorescence intensity analysis

**Fig. 5:**
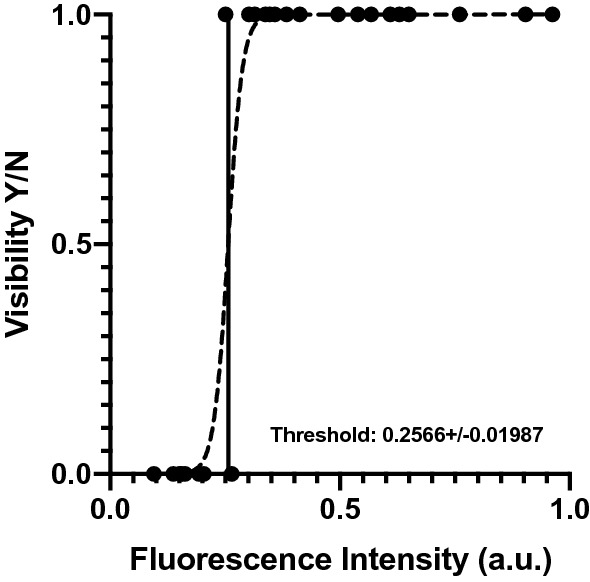
Logistic regression defining the fluorescence visibility threshold. Logistic regression of the mean of the fluorescence intensity of each image and its relative binary classification (visible (Y) and not visible (N))

The human eye visibility threshold measured 0.25 ± 0.02 a.u. (Fig. 5). The mean fluorescence of ICG tattooing in the stomach at T0 (0.55 ± 0.21 a.u.) was significantly higher than at T1 (0.23 ± 0.06 a.u., *p* value = 0.001) and at T2 (0.18 ± 0.02 a.u., *p* value = 0.0004). No difference was found between T1 and T2.

**Fig. 6:**
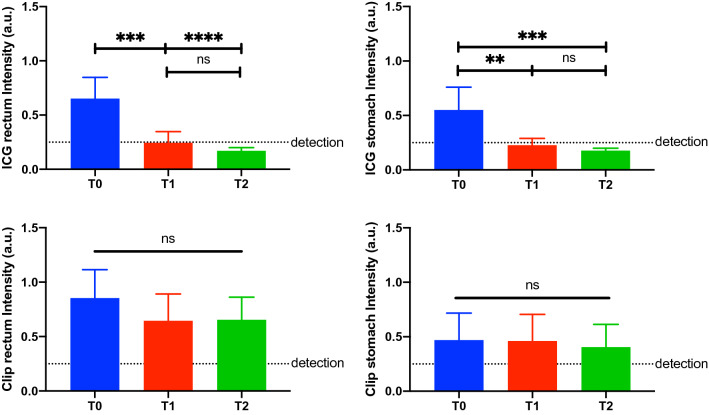
Quantitative fluorescence intensity analysis. Quantitative analysis of the ICG tattooing (above) and the clip markings (below), both rectum (left) and stomach (right) are displayed. Interestingly, the tattoo showed a short durability over time, whereas the clip marking showed a stable intensity. The maximal intensity of both methods is higher in the rectum than in the stomach, since the first has thinner walls than the latter. The dotted line shows the human fluorescence detectability threshold

Similarly, the mean fluorescence intensity of ICG tattooing in the rectum at T0 (0.65 ± 0.19 a.u.) was statistically significantly higher than at T1 (0.24 ± 0.1 a.u., *p* value = 0.0002) and at T2 (0.17 ± 0.03 a.u., *p* value < 0.0001). The intensity at T1 and T2 did not show any difference (Fig. 6).

The mean fluorescence intensity of the clip in the stomach and in the rectum at T0 (0.47 ± 0.25 a.u. and 0.85 ± 0.26 a.u., respectively) did not differ from T1 (0.46 ± 0.25 a.u. and 0.65 ± 0.26 a.u., respectively) and T2 (0.4 ± 0.21 a.u. and 0.65 ± 0.21 a.u., respectively).

### Human anatomical specimen experiment

**Fig. 7:**
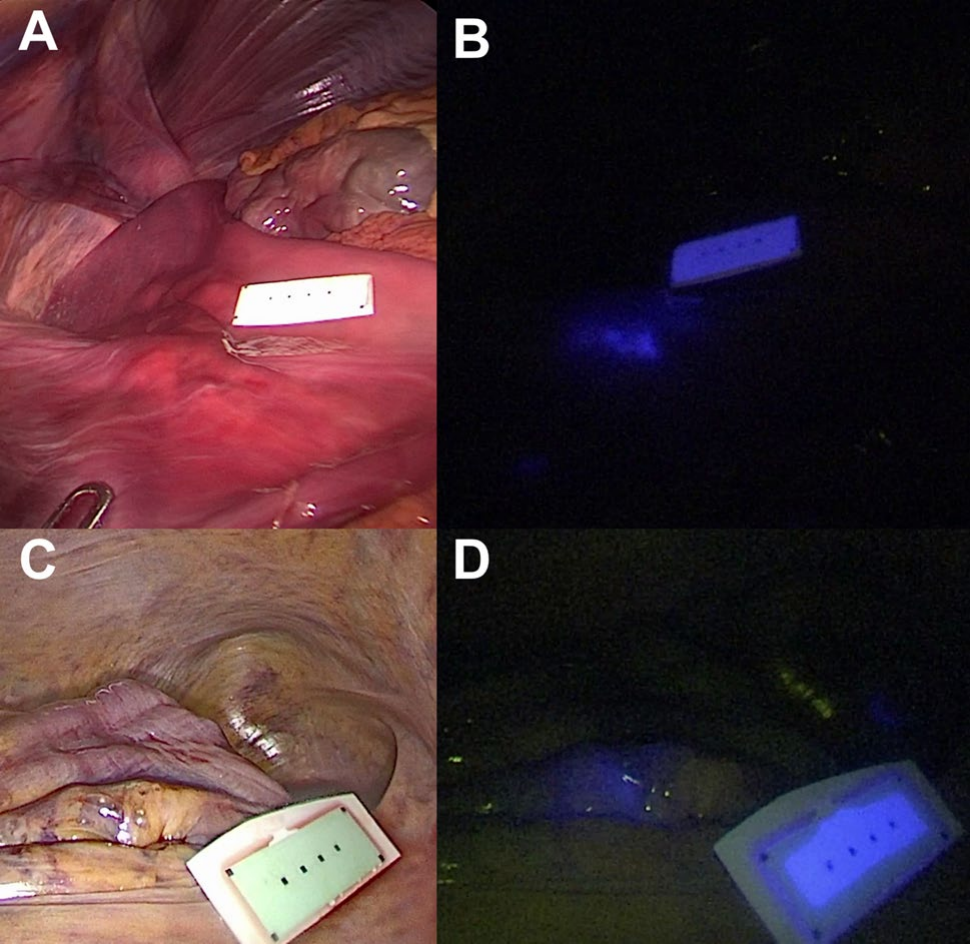
Fluorescent clip marking in the human anatomical specimen. Laparoscopic white light (left) and NIR imaging (right) mode visualization of the FOSC into the cadaver’s stomach (**A**, **B**) and sigmoid colon (**C**, **D**)

The fluorescent clips placed in the colon and in the stomach were clearly visible using the NIR mode of the laparoscopic camera (Fig. 7 and Videoclip).

## Discussion

In the current survival study, we propose a fluorescently coated over the scope clip as an alternative endoscopic preoperative marking method. The over the scope clip is a robust clip, which can remain in place for las long as 60 days after its application [25] and with an extremely low complications rate [26]. We show that our coated FOSC remains visible longer and is as safe as ICG tattooing. There was no statistically significant decrease in the clip’s fluorescence intensity over time, as observed in the quantitative analysis, and the fluorescence remained always above the human visibility threshold. Additionally, the FOSC was also easily visible through the human colon and gastric walls.

The main advantage of ICG over non-fluorescent dyes (India ink and SPOT™) is that, in cases of intraperitoneal spillage, the surgical dissection planes are potentially less compromised under white light. For this reason, several authors have recently successfully used ICG as an efficient and safe alternative gastrointestinal tattooing method in clinical trials [13–18]. However, in most of those trials, the endoscopic ICG tattooing was performed 1 to 3 days preoperatively which is clinically improbable. In one study, Watanabe et al*.* [17] extended the tattooing/surgery interval to as long as 17 days. The authors reported decreasing visibility of the ICG tattoos that reached 0% by the 10th day. The overall visibility was 98.7% until the 7th day, so they concluded that the optimal endoscopy/surgery interval should be a maximum of 1 week to ensure a high intraoperative tattooing detectability.

Initially, in our study, laparoscopic follow-up T1 was scheduled for POD 6. This interval was respected in 3 animals, and yet we found a 0% ICG tattoo detection rate in both organs. As a result, we decided to advance the T1 follow-up to POD 4 for the following 3 animals. By doing so, the overall T1 ICG tattoo detection rate increased to 33.3% for the stomach and 50% for the colon. The longer duration found by Watanabe et al. may be explained by the higher dose of ICG (2.5 mg/mL) administered, whereas in this experimental study we used a lower dose (0.05 mg/mL).

Additionally, Watanabe et al*.* used a PINPOINT® (Novadaq Technologies Inc., United States) NIR camera for the laparoscopic tattoo detection. This NIR imager produces pictures with a superior fluorescent signal contrast than the imaging system used in our study [27]. Currently, there is no consensus on the ICG concentration required for submucosal GI tattooing and the literature has a large dose variability [13–18]. The ICG dosing chosen for our study was the same utilized in the successful work by Ushimaru et al*.* on gastric tumor tattooing [14]. This report is the largest clinical trial using ICG as GI tract tattooing mean and employed the same laparoscopic NIR optical system of our work.

Although ICG tattooing has been shown to be possible, provided that the tattooing/surgery interval was short, the high diffusion in the submucosal space, reported to be up to 7 cm [14], is also also an issue. While the ICG diffusion rate was not objectively quantified in our set-up, we noted a significant dye diffusion from the injection site even at T0, as the porcine GI tract was tattooed under direct laparoscopic visualization. Theoretically, this could limit the accuracy of tumor localization, especially for small endoluminal lesions undergoing function-saving precise resections. The fluorescent signal of the FOSC was shown to be very precise in our experiments and was limited to the shape of the clip itself.

Other groups have previously described preoperative marking of gastrointestinal lesions using fluorescent clips [28–30]. In these studies, the authors could successfully identify the endoluminal clips from the serosal side using NIR cameras. However, these were based on traditional through-the-scope endoclips, and could be expected to fall off relatively early [12]. These studies were all acute, therefore this potential limitation could not be assessed.

Because of their small size and tendency to fall off, when endoclips are used to mark GI lesions, multiple clips are often necessary [31, 32]. This potentially increases their success, but simultaneously increases costs. Given the ability of over the scope clips to anchor to the colonic/gastric wall for up to 2 months [25], the placement of a single fluorescent OSC in proximity of the lesion could be sufficient to achieve successful localization.

Current over the scope clips are rather expensive and this might limit their clinical acceptability as preoperative endoscopic markers. However, the interval between diagnosis and surgical resection of colorectal and gastric cancer is on average 4–6 weeks for colorectal and gastric cancers [33]. Consequently, the use of a long-lasting FOSC during the diagnostic endoscopy, might spare additional preoperative endoscopic marking procedures closer to surgery.

During the study one serious complication of a small bowel perforation occurred. This was probably due to traumatic manipulation of the bowel and seemed to be unrelated to the clips or tattooing injection sites. In this animal, in spite of the increased tissue thickness from the fecal peritonitis, the clips were perfectly visible on the gastric and colonic sites and were, therefore, included in the quantitative fluorescence analysis.

The merits of our study lie in the innovative solution proposed, in the survival comparative design of the animal experiments, together with the objective quantitative fluorescence analysis. The proof-of-the-concept in the human cadaver experiment represents an added value to reinforce a possible future clinical translation.

Our work has some limits. The survival period was too short to truly assess the long-term durability of the fluorescent clips.

At the current stage the FOSC needs to be prepared by immerging the clip 3 times in the NICE coating, at least 30 min prior to the procedures. This limits their usability in the clinical setting. To overcome this drawback, one could imagine ready-to-use commercially available FOSCs in the future.

The expense of the clips might limit their use as well, however increasing indications for their use might induce a progressive price decrease. Further studies to explore the cost-effectiveness of our novel endoscopic tumor marking method would be helpful.

Another downside of using FOSC as a marking device is the fact that once a lesion has been discovered, the scope has to be removed in order to mount the clip onto the scope. This also slightly increasing the diameter of the scope’s tip which might be troublesome, especially when dealing with high colonic lesions. In cases where the lesion is known, the procedure could be performed with the OSC system already mounted on the scope, hence reducing the preoperative endoscopic procedure time.

In conclusion, we proposed an innovative, precise, and durable GI lesion marking tool and show its superiority in comparison to ICG submucosal tattooing in the porcine model. Furthermore, the fluorescent over-the-scope clip was perfectly detectable in a human cadaver’s GI tract.

The NICE coating is currently undergoing regulatory approval for human use and at this stage, we plan to initiate a clinical trial to assess the utility of this new approach in patients.

## Electronic supplementary material

Below is the link to the electronic supplementary material.

Supplementary file1 (MP4 194040 kb)

Supplementary file2 (TIFF 2073 kb) Fluorescent clip and delivery system. The clip mounted onto the delivery system is visible under white light (A) and under near-infrared light (B). The clip in its closed position, once released from the delivery system, is shown under white light (C) and near-infrared light (D).
