# Supplementary figures and images for: Preoperative endoscopic marking of the gastrointestinal tract using fluorescence imaging: submucosal indocyanine green tattooing versus a novel fluorescent over-the-scope clip in a survival experimental study
Source: Surg Endosc. 2020 Sep 28;35(9):5115–23. doi: 10.1007/s00464-020-07999-2 (PMC8346416; doi:10.1007/s00464-020-07999-2)

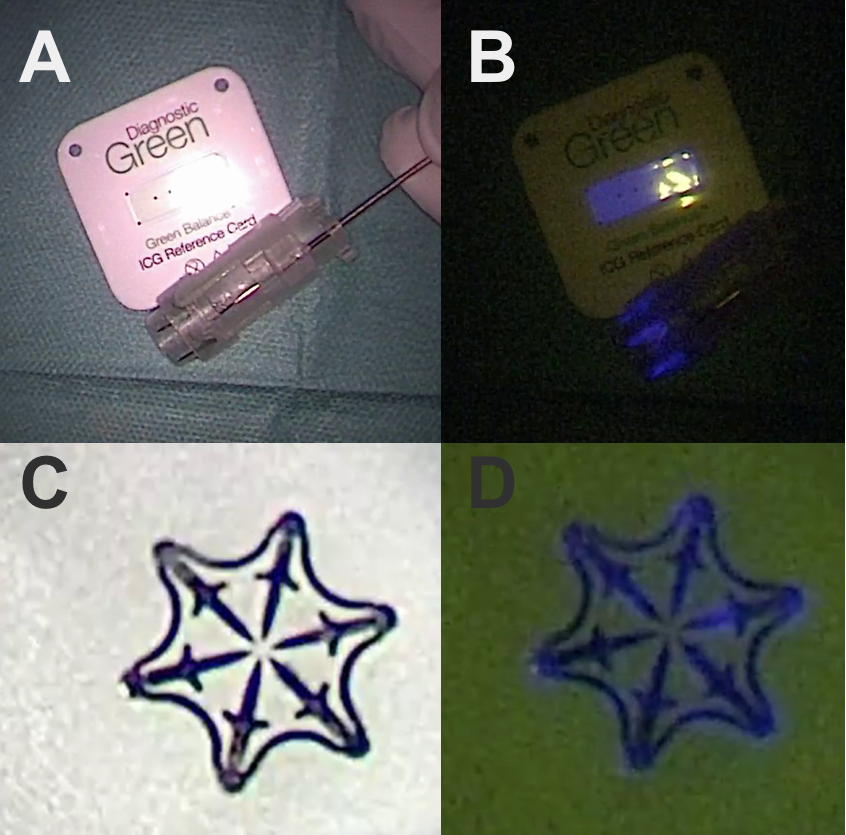

Supplement: Supplementary file 2 — Supplementary file2 (TIFF 2073 kb) Fluorescent clip and delivery system. The clip mounted onto the delivery system is visible under white light (A) and under near-infrared light (B). The clip in its closed position, once released from the delivery system, is shown under white light (C) and near-infrared light (D). [file 464_2020_7999_MOESM2_ESM.tiff]
